# Supplementary material for: Identifying Factors That Predict Behavioral Intention to Stay under Lockdown during the SARS-CoV-2 Pandemic Using a Structural Equation Model
Source: Int J Environ Res Public Health. 2022 Feb 27;19(5):2757. doi: 10.3390/ijerph19052757 (PMC8910594; doi:10.3390/ijerph19052757)
Supplement: Supplementary file 1 [file ijerph-19-02757-s001.zip › ijerph-1616037-supplementary.pdf]

Investigación sobre la Cuarentena

El presente cuestionario forma parte de una investigación que se realiza en todo México desde la Facultad de Ingeniería y Negocios Guadalupe Victoria de la Universidad Autónoma de Baja California. El investigador responsable es el Dr. Joaquín Alberto Padilla Bautista, su correo joaquin.padilla@uabc.edu.mx. Esta investigación busca conocer cómo las personas de diferentes ciudades afronta la cuarentena y sus repercusiones en la salud física y psicológica. Su objetivo es contar con conocimiento que pueda ayudar a todas las personas.

La información que brindes es CONFIDENCIAL y ANÓNIMA y sólo se utilizará para fines de investigación. No hay respuestas correctas o incorrectas, por lo que le pedimos que conteste todas las preguntas con la mayor sinceridad posible. Gracias por su atención y tiempo.

---

*Obligatorio

1. 1.- ¿Brindas tu consentimiento para participar en esta investigación? *

   Marca solo un óvalo.

   - Sí. Brindo mi consentimiento y declaro ser mayor de 18 años. *Ir a la pregunta 2*
   - No

Demográficos

2. Si quiere recibir información sobre esta aplicación por favor proporcione un correo electrónico. Recuerde que en todo momento se mantendrá el anonimato.

   ____

3. 1.- Soy: *

   Marca solo un óvalo.

   - Hombre
   - Mujer
   - Otros: ____________________

4. 2.- Mi edad en años es: *

5. 3.- ¿Cuál es tu ocupación? *

6. 4.- ¿En qué PAÍS vives actualmente? *

7. 4.1- ¿En qué CIUDAD vives actualmente? *

8. 5.- ¿Cuáles es tu escolaridad terminada? *

Marca solo un óvalo.

- Primaria
- Secundaria
- Técnico / bachillerato
- Licenciatura
- Posgrado

9. 6.- ¿Cómo consideras que es tu salud actualmente? *

Marca solo un óvalo.

- Muy mala
- Mala
- Regular
- Buena
- Muy buena

10. 7.- ¿Cómo consideras tu condición física actualmente? *

Marca solo un óvalo.

- Muy mala
- Mala
- Regular
- Buena
- Muy buena

11. 8.- ¿Cómo consideras tu nivel de estrés actualmente? *

Marca solo un óvalo.

- Muy bajo
- Bajo
- Normal
- Alto
- Muy alto

12. 9.- ¿Cuál es tu peso en kilogramos? *

13. 10.- ¿Cuál es tu estatura en metros? *

14. 11.- En promedio ¿Cuántas horas duermes por noche? *

15. 12.- En promedio ¿Cuál es la calidad de tus horas de sueño? *

Marca solo un óvalo.

- Muy mala
- Mala
- Regular
- Buena
- Muy buena

16. 13.- En promedio ¿Cuántas horas semanalmente dedicas al ejercicio? *

17. 14.- ¿Cuántas comidas haces al día? *

18. 15.- Cuando comes, ¿Te gusta comer? *

Marca solo un óvalo.

- Poco
- Normal
- Mucho

19. 16.- ¿Padeces actualmente de alguna enfermedad? *

Marca solo un óvalo.

- Sí
- No

20. ¿Cuál?

21. 17.- ¿Cuánto riesgo de contagio sientes que hay en tu CIUDAD? *

Marca solo un óvalo.

- Nada
- Muy bajo
- Bajo
- Normal
- Alto
- Muy alto

22. 18.- ¿Cuánto riesgo de contagio siente que hay en tu HOGAR? *

Marca solo un óvalo.

- Nada
- Muy bajo
- Bajo
- Normal
- Alto
- Muy alto

23. 19.- ¿Conoces a personas contagiadas por CORONAVIRUS / COVID -19? *

Marca solo un óvalo.

- Sí
- No
- No tengo la certeza

24. ¿Quién o quienes?

25. 20.- En tu casa vives: *

Marca solo un óvalo.

- Sola / solo
- Con mi familia
- Con mi pareja
- Con mi pareja y familia
- Con mis hijos
- Con mis hijos y pareja
- Con mis hijos, pareja y familia
- Otros: ____________

26. 21.- ¿Cuántos hijos tienes?

27. 22.- ¿Cuántas personas dependen económicamente de ti?

28. 23.- Aproximadamente ¿Cuantos días llevas en cuarentena? *

29. 24.- ¿Que tan fácil a sido la cuarentena para ti? *

Marca solo un óvalo.

- Muy facil
- Facil
- Normal
- Difícil
- Muy difícil

30. 25.- ¿Crees que podrás realizar lo que resta de la cuarentena? *

Marca solo un óvalo.

- Sí
- No
- Tal vez

31. 26.- ¿Actualmente te encuentras en alguno tipo de terapia psicológica? *

Marca solo un óvalo.

- Sí
- No

32. 27.- Si tu respuesta fue sí, ¿Que tipo de terapia tomas?

En esta sección seleccione la opción que mejor describa su situación.

33. Durante la cuarentena: *

Marca solo un óvalo.

- Salgo MÁS que antes.
- Salgo IGUAL que antes y hago mi vida normal.
- He tomado medidas de precaución, pero sigo saliendo.
- He dejado de salir, pero familiares o amigos entran y salen de casa.
- Nadie sale de casa, ni se aceptan visitas y solo Yo o un familiar sale por alimentos.
- Ninguna persona entra o sale, todo se pide a domicilio.
- Estoy en cuarentena total, nada entra o sale, me sostengo con recursos adquiridos previamente.

Mantenerme en cuarentena es:

34. 1.- Importante *

Marca solo un óvalo.

	1	2	3	4	5	
Nada						Muchísimo

35. 2.- Estresante *

Marca solo un óvalo.

	1	2	3	4	5	
Nada	⬭	⬭	⬭	⬭	⬭	Muchísimo

36. 3.- Un momento de reflexión *

Marca solo un óvalo.

	1	2	3	4	5	
Nada	⬭	⬭	⬭	⬭	⬭	Muchísimo

37. 4.- Inútil *

Marca solo un óvalo.

	1	2	3	4	5	
Nada	⬭	⬭	⬭	⬭	⬭	Muchísimo

38. 5.- Oportunidad de crecimiento *

Marca solo un óvalo.

	1	2	3	4	5	
Nada	⬭	⬭	⬭	⬭	⬭	Muchísimo

39. 6.- Bueno *

Marca solo un óvalo.

	1	2	3	4	5	
Nada	◯	◯	◯	◯	◯	Muchísimo

40. 7.- Aburrido *

Marca solo un óvalo.

	1	2	3	4	5	
Nada	◯	◯	◯	◯	◯	Muchísimo

41. 8.- Inteligente *

Marca solo un óvalo.

	1	2	3	4	5	
Nada	◯	◯	◯	◯	◯	Muchísimo

42. 9.- Malo *

Marca solo un óvalo.

	1	2	3	4	5	
Nada	◯	◯	◯	◯	◯	Muchísimo

43. 10.- Cansado *

Marca solo un óvalo.

	1	2	3	4	5	
Nada						Muchísimo

44. 11.- Desesperante *

Marca solo un óvalo.

	1	2	3	4	5	
Nada						Muchísimo

45. 12.- Monótono *

Marca solo un óvalo.

	1	2	3	4	5	
Nada						Muchísimo

46. 13.- Descanso *

Marca solo un óvalo.

	1	2	3	4	5	
Nada						Muchísimo

47. 14.- Sufrimiento *

Marca solo un óvalo.

	1	2	3	4	5	
Nada	◯	◯	◯	◯	◯	Muchísimo

48. 15.- Desafiante *

Marca solo un óvalo.

	1	2	3	4	5	
Nada	◯	◯	◯	◯	◯	Muchísimo

49. 16.- Angustiante *

Marca solo un óvalo.

	1	2	3	4	5	
Nada	◯	◯	◯	◯	◯	Muchísimo

50. 17.- Responsable *

Marca solo un óvalo.

	1	2	3	4	5	
Nada	◯	◯	◯	◯	◯	Muchísimo

51. 18.- Necesario *

Marca solo un óvalo.

	1	2	3	4	5	
Nada	◯	◯	◯	◯	◯	Muchísimo

Mis seres queridos (Familia, amigos o pareja) en esta cuarentena...

52. 1. me apoyan para que la lleve acabo. *

Marca solo un óvalo.

	1	2	3	4	5	
Nada	◯	◯	◯	◯	◯	Muchisimo

53. 2. piensan que es correcto quedarme en casa. *

Marca solo un óvalo.

	1	2	3	4	5	
Nada	◯	◯	◯	◯	◯	Muchisimo

54. 3. piensan que tengo que salir a trabajar. *

Marca solo un óvalo.

	1	2	3	4	5	
Nada	◯	◯	◯	◯	◯	Muchisimo

55. 4. se preocupan por mi salud. *

Marca solo un óvalo.

	1	2	3	4	5	
Nada	◯	◯	◯	◯	◯	Muchisimo

56. 5. me piden que no salga. *

Marca solo un óvalo.

	1	2	3	4	5	
Nada	◯	◯	◯	◯	◯	Muchisimo

57. 6. creen que exagero al cuidarme tanto. *

Marca solo un óvalo.

	1	2	3	4	5	
Nada	◯	◯	◯	◯	◯	Muchisimo

58. 7. creen que entre menos salga menor será el riesgo de enfermarme. *

Marca solo un óvalo.

	1	2	3	4	5	
Nada	◯	◯	◯	◯	◯	Muchisimo

59. 8. están de acuerdo con que realice la cuarentena. *

Marca solo un óvalo.

	1	2	3	4	5	
Nada	◯	◯	◯	◯	◯	Muchisimo

60. 9. me piden que vaya a sus casas. *

Marca solo un óvalo.

	1	2	3	4	5	
Nada	◯	◯	◯	◯	◯	Muchisimo

61. 10. me critican por no hacerla. *

Marca solo un óvalo.

	1	2	3	4	5	
Nada	◯	◯	◯	◯	◯	Muchisimo

62. 11. piensan que es mi obligación no salir. *

Marca solo un óvalo.

	1	2	3	4	5	
Nada	◯	◯	◯	◯	◯	Muchisimo

63. 12. se enojan porque NO salgo a trabajar. *

Marca solo un óvalo.

	1	2	3	4	5	
Nada	⬭	⬭	⬭	⬭	⬭	Muchisimo

64. 13. dicen que es importante hacerla por seguridad de todos. *

Marca solo un óvalo.

	1	2	3	4	5	
Nada	⬭	⬭	⬭	⬭	⬭	Muchisimo

65. 14. piensan que es mi problema. *

Marca solo un óvalo.

	1	2	3	4	5	
Nada	⬭	⬭	⬭	⬭	⬭	Muchisimo

66. 15. me piden que cumpla con mi jornada laboral. *

Marca solo un óvalo.

	1	2	3	4	5	
Nada	⬭	⬭	⬭	⬭	⬭	Muchisimo

Durante esta cuarentena...

67. 1. tengo compañía. *

Marca solo un óvalo.

	1	2	3	4	5	
No estoy de acuerdo						Estoy muy de acuerdo

68. 2. en mi casa nos llevamos bien. *

Marca solo un óvalo.

	1	2	3	4	5	
No estoy de acuerdo						Estoy muy de acuerdo

69. 3. mi familia está segura. *

Marca solo un óvalo.

	1	2	3	4	5	
No estoy de acuerdo						Estoy muy de acuerdo

70. 4. puedo arreglar los problemas que se presenten. *

Marca solo un óvalo.

	1	2	3	4	5	
No estoy de acuerdo						Estoy muy de acuerdo

71. 5. cuento con ahorros para salir adelante. *

Marca solo un óvalo.

	1	2	3	4	5	
No estoy de acuerdo						Estoy muy de acuerdo

72. 6. tengo certidumbre de lo que está pasando. *

Marca solo un óvalo.

	1	2	3	4	5	
No estoy de acuerdo						Estoy muy de acuerdo

73. 7. sé cómo cuidar de mí. *

Marca solo un óvalo.

	1	2	3	4	5	
No estoy de acuerdo						Estoy muy de acuerdo

74. 8. sé que mi trabajo está seguro. *

Marca solo un óvalo.

	1	2	3	4	5	
No estoy de acuerdo						Estoy muy de acuerdo

75. 9. tengo un cuerpo sano. *

Marca solo un óvalo.

	1	2	3	4	5	
No estoy de acuerdo						Estoy muy de acuerdo

76. 10. tengo actividades para no aburrirme. *

Marca solo un óvalo.

	1	2	3	4	5	
No estoy de acuerdo						Estoy muy de acuerdo

77. 11. cuido a mi familia. *

Marca solo un óvalo.

	1	2	3	4	5	
No estoy de acuerdo						Estoy muy de acuerdo

78. 12. Podre manejar cualquier situación imprevista. *

Marca solo un óvalo.

	1	2	3	4	5	
No estoy de acuerdo						Estoy muy de acuerdo

79. 13. Se que tengo las habilidades necesarias para salir adelante. *

Marca solo un óvalo.

	1	2	3	4	5	
No estoy de acuerdo	◯	◯	◯	◯	◯	Estoy muy de acuerdo

80. 14. cuento con un apoyo económico. *

Marca solo un óvalo.

	1	2	3	4	5	
No estoy de acuerdo	◯	◯	◯	◯	◯	Estoy muy de acuerdo

81. 15. tengo contacto con mis amigos. *

Marca solo un óvalo.

	1	2	3	4	5	
No estoy de acuerdo	◯	◯	◯	◯	◯	Estoy muy de acuerdo

82. 16. tengo una buena conexión a internet. *

Marca solo un óvalo.

	1	2	3	4	5	
No estoy de acuerdo	◯	◯	◯	◯	◯	Estoy muy de acuerdo

83. 17. puedo aplazar los pagos de servicios (Luz, agua, etc.). *

Marca solo un óvalo.

	1	2	3	4	5	
No estoy de acuerdo	◯	◯	◯	◯	◯	Estoy muy de acuerdo

84. 18. me despidieron de mi trabajo. *

Marca solo un óvalo.

	1	2	3	4	5	
No estoy de acuerdo	◯	◯	◯	◯	◯	Estoy muy de acuerdo

85. 18.1 por favor para saber que esta leyendo selecciona el numero dos. *

Marca solo un óvalo.

	1	2	3	4	5	
No estoy de acuerdo	◯	◯	◯	◯	◯	Estoy muy de acuerdo

86. 19. en mi casa tengo un lugar para mí solo. *

Marca solo un óvalo.

	1	2	3	4	5	
No estoy de acuerdo	◯	◯	◯	◯	◯	Estoy muy de acuerdo

87. 20. puedo trabajar desde casa. *

Marca solo un óvalo.

	1	2	3	4	5	
No estoy de acuerdo	⬭	⬭	⬭	⬭	⬭	Estoy muy de acuerdo

88. 21. me entregan alimentos u otras cosas a domicilio. *

Marca solo un óvalo.

	1	2	3	4	5	
No estoy de acuerdo	⬭	⬭	⬭	⬭	⬭	Estoy muy de acuerdo

89. 22. sigo saliendo a buscar ingresos. *

Marca solo un óvalo.

	1	2	3	4	5	
No estoy de acuerdo	⬭	⬭	⬭	⬭	⬭	Estoy muy de acuerdo

90. 23. mi familia necesita que salga. *

Marca solo un óvalo.

	1	2	3	4	5	
No estoy de acuerdo	⬭	⬭	⬭	⬭	⬭	Estoy muy de acuerdo

91. 24. tengo alimento suficiente. *

Marca solo un óvalo.

	1	2	3	4	5	
No estoy de acuerdo						Estoy muy de acuerdo

92. 25. tengo el dinero suficiente. *

Marca solo un óvalo.

	1	2	3	4	5	
No estoy de acuerdo						Estoy muy de acuerdo

93. 26. necesito asistencia médica que me obliga a salir. *

Marca solo un óvalo.

	1	2	3	4	5	
No estoy de acuerdo						Estoy muy de acuerdo

Yo...

94. 1. Me siento triste y sin ánimo. *

Marca solo un óvalo.

	1	2	3	4	5	
La MENOR parte del tiempo						La MAYOR parte del tiempo

95. 2. Me siento mejor por las mañanas. *

Marca solo un óvalo.

	1	2	3	4	5	
La MENOR parte del tiempo						La MAYOR parte del tiempo

96. 3. Por momentos caigo en llanto o tengo ganas de llorar. *

Marca solo un óvalo.

	1	2	3	4	5	
La MENOR parte del tiempo						La MAYOR parte del tiempo

97. 4. Me alimento tanto como solía hacerlo. *

Marca solo un óvalo.

	1	2	3	4	5	
La MENOR parte del tiempo						La MAYOR parte del tiempo

98. 5. Tengo problemas para dormir por la noche. *

Marca solo un óvalo.

	1	2	3	4	5	
La MENOR parte del tiempo						La MAYOR parte del tiempo

99. 6. Sigo disfrutando el sexo. *

Marca solo un óvalo.

	1	2	3	4	5	
La MENOR parte del tiempo						La MAYOR parte del tiempo

100. 7. Me doy cuenta de que estoy perdiendo peso. *

Marca solo un óvalo.

	1	2	3	4	5	
La MENOR parte del tiempo						La MAYOR parte del tiempo

101. 8. Mi mente esta tan clara como siempre. *

Marca solo un óvalo.

	1	2	3	4	5	
La MENOR parte del tiempo						La MAYOR parte del tiempo

102. 9. Tengo problemas de estreñimiento. *

Marca solo un óvalo.

	1	2	3	4	5	
La MENOR parte del tiempo						La MAYOR parte del tiempo

103. 10. Encuentro fácil hacer las mismas tareas de siempre. *

Marca solo un óvalo.

	1	2	3	4	5	
La MENOR parte del tiempo						La MAYOR parte del tiempo

104. 11. Mi corazón late más rápido de lo normal. *

Marca solo un óvalo.

	1	2	3	4	5	
La MENOR parte del tiempo						La MAYOR parte del tiempo

105. 12. Me resulta fácil tomar decisiones. *

Marca solo un óvalo.

	1	2	3	4	5	
La MENOR parte del tiempo						La MAYOR parte del tiempo

106. 13. Me canso sin razón. *

Marca solo un óvalo.

	1	2	3	4	5	
La MENOR parte del tiempo						La MAYOR parte del tiempo

107. 14. Siento que soy útil y necesario. *

Marca solo un óvalo.

	1	2	3	4	5	
La MENOR parte del tiempo	⬭	⬭	⬭	⬭	⬭	La MAYOR parte del tiempo

108. 15. Siento angustia. *

Marca solo un óvalo.

	1	2	3	4	5	
La MENOR parte del tiempo	⬭	⬭	⬭	⬭	⬭	La MAYOR parte del tiempo

109. 16. Tengo esperanzas en el futuro. *

Marca solo un óvalo.

	1	2	3	4	5	
La MENOR parte del tiempo	⬭	⬭	⬭	⬭	⬭	La MAYOR parte del tiempo

110. 17. Estoy más irritable de lo normal. *

Marca solo un óvalo.

	1	2	3	4	5	
La MENOR parte del tiempo	⬭	⬭	⬭	⬭	⬭	La MAYOR parte del tiempo

111. 18. Tengo una vida llena de plenitud. *

Marca solo un óvalo.

	1	2	3	4	5	
La MENOR parte del tiempo	◯	◯	◯	◯	◯	La MAYOR parte del tiempo

112. 19. Siento que los demás estarían mejor si yo estuviera muerto. *

Marca solo un óvalo.

	1	2	3	4	5	
La MENOR parte del tiempo	◯	◯	◯	◯	◯	La MAYOR parte del tiempo

113. 20. Sigo disfrutando de las cosas que solía hacer. *

Marca solo un óvalo.

	1	2	3	4	5	
La MENOR parte del tiempo	◯	◯	◯	◯	◯	La MAYOR parte del tiempo

114. 1. Siento más ansiedad y nerviosismo de lo normal. *

Marca solo un óvalo.

	1	2	3	4	5	
La MENOR parte del tiempo	◯	◯	◯	◯	◯	La MAYOR parte del tiempo

115. 2. Tengo miedo sin algún motivo. *

Marca solo un óvalo.

	1	2	3	4	5	
La MENOR parte del tiempo	⬭	⬭	⬭	⬭	⬭	La MAYOR parte del tiempo

116. 3. Me altero o siento pánico fácilmente. *

Marca solo un óvalo.

	1	2	3	4	5	
La MENOR parte del tiempo	⬭	⬭	⬭	⬭	⬭	La MAYOR parte del tiempo

117. 4. Siento como si me estuviera cayendo a pedazos. *

Marca solo un óvalo.

	1	2	3	4	5	
La MENOR parte del tiempo	⬭	⬭	⬭	⬭	⬭	La MAYOR parte del tiempo

118. 5. Siento que todo está bien y que no pasara nada malo. *

Marca solo un óvalo.

	1	2	3	4	5	
La MENOR parte del tiempo	⬭	⬭	⬭	⬭	⬭	La MAYOR parte del tiempo

119. 6. Mis brazos y piernas tiemblan. *

Marca solo un óvalo.

	1	2	3	4	5	
La MENOR parte del tiempo	⬭	⬭	⬭	⬭	⬭	La MAYOR parte del tiempo

120. 7. Tengo dolores de cabeza, cuello o espalda. *

Marca solo un óvalo.

	1	2	3	4	5	
La MENOR parte del tiempo	⬭	⬭	⬭	⬭	⬭	La MAYOR parte del tiempo

121. 8. Me siento débil y me canso fácilmente. *

Marca solo un óvalo.

	1	2	3	4	5	
La MENOR parte del tiempo	⬭	⬭	⬭	⬭	⬭	La MAYOR parte del tiempo

122. 9. Me siento en tranquilidad y me es fácil estar en calma. *

Marca solo un óvalo.

	1	2	3	4	5	
La MENOR parte del tiempo	⬭	⬭	⬭	⬭	⬭	La MAYOR parte del tiempo

123. 10. Siento que mi corazón late rápido. *

Marca solo un óvalo.

	1	2	3	4	5	
La MENOR parte del tiempo	◯	◯	◯	◯	◯	La MAYOR parte del tiempo

124. 11. Sufro de mareos o vértigo. *

Marca solo un óvalo.

	1	2	3	4	5	
La MENOR parte del tiempo	◯	◯	◯	◯	◯	La MAYOR parte del tiempo

125. 12. Me desmayo o siento que voy a desmayarme. *

Marca solo un óvalo.

	1	2	3	4	5	
La MENOR parte del tiempo	◯	◯	◯	◯	◯	La MAYOR parte del tiempo

126. 13. Puedo inhalar y exhalar con tranquilidad. *

Marca solo un óvalo.

	1	2	3	4	5	
La MENOR parte del tiempo	◯	◯	◯	◯	◯	La MAYOR parte del tiempo

127. 14. Siento que se entumecen u hormiguean mis dedos. *

Marca solo un óvalo.

	1	2	3	4	5	
La MENOR parte del tiempo						La MAYOR parte del tiempo

128. 15. Sufro de dolores de estómago o indigestión. *

Marca solo un óvalo.

	1	2	3	4	5	
La MENOR parte del tiempo						La MAYOR parte del tiempo

129. 16. Tengo que orinar constantemente. *

Marca solo un óvalo.

	1	2	3	4	5	
La MENOR parte del tiempo						La MAYOR parte del tiempo

130. 17. Mis manos usualmente están cálidas y secas. *

Marca solo un óvalo.

	1	2	3	4	5	
La MENOR parte del tiempo						La MAYOR parte del tiempo

131. 17.1 Por favor para saber que estas leyendo selecciona el numero cuatro. *

Marca solo un óvalo.

	1	2	3	4	5	
La MENOR parte del tiempo	◯	◯	◯	◯	◯	La MAYOR parte del tiempo

132. 18. Siento mi cara caliente y sonrojada. *

Marca solo un óvalo.

	1	2	3	4	5	
La MENOR parte del tiempo	◯	◯	◯	◯	◯	La MAYOR parte del tiempo

133. 19. Duermo fácilmente y descanso bien por las noches. *

Marca solo un óvalo.

	1	2	3	4	5	
La MENOR parte del tiempo	◯	◯	◯	◯	◯	La MAYOR parte del tiempo

134. 20. Tengo pesadillas. *

Marca solo un óvalo.

	1	2	3	4	5	
La MENOR parte del tiempo	◯	◯	◯	◯	◯	La MAYOR parte del tiempo

Para terminar.

135. 1. Pensando en el jefe o jefa de hogar, ¿cuál fue el último año de estudios que aprobó en la escuela? *

Marca solo un óvalo.

- Sin estudios
- Preescolar
- Primaria Incompleta
- Primaria Completa
- Secundaria Incompleta
- Secundaria Completa
- Preparatoria Incompleta
- Preparatoria Completa
- Licenciatura Incompleta
- Licenciatura Completa
- Posgrado

136. 2. ¿Cuántos baños completos con regadera y W.C. (excusado) hay en esta vivienda? *

Marca solo un óvalo.

- 0
- 1
- 2 ó más

137. 3. ¿Cuántos automóviles o camionetas tienen en su hogar, incluyendo camionetas cerradas, o con cabina o caja? *

Marca solo un óvalo.

- 0
- 1
- 2 ó más

138. 4. Sin tomar en cuenta la conexión móvil que pudiera tener desde algún celular ¿este hogar cuenta con Internet? *

Marca solo un óvalo.

- No tiene
- Sí tiene

139. 5. De todas las personas de 14 años o más que viven en el hogar, ¿cuántas trabajaron en el último mes? *

Marca solo un óvalo.

- 0
- 1
- 2
- 3
- 4 ó más

140. 6. En esta vivienda, ¿cuántos cuartos se usan para dormir, sin contar pasillos ni baños? *

Marca solo un óvalo.

- 0
- 1
- 2
- 3
- 4 ó más

141. ¿Has buscado algún tipo de ayuda psicológica? *

Marca solo un óvalo.

- Sí, y la he conseguido
- Sí, y NO la he conseguido.
- No
- Otros: ____________________

142. Para nosotros es muy importante dar un seguimiento durante esta cuarentena, por favor, déjanos tu correo si estas de acuerdo en participan en la segunda aplicación de este mismo cuestionario. Tu correo se usara solo para este fin y una sola vez. Muchas Gracias.

____

¿Tienes personas mayores viviendo contigo? ayúdanos a aplicarles la encuesta, así también sabrás como se sienten. ¡Muchas gracias por tu colaboración!

Si necesitas Atención psicológica, puedes llamar al consejo Ciudad de México, las 24 horas del día el teléfono es : 55 55 33 55 33

Google no creó ni aprobó este contenido.

Google Formularios
